# Graphene oxide/reduced graphene oxide films as protective barriers on lead against differential aeration corrosion induced by water drops†

**DOI:** 10.1039/d0na00212g

**Published:** 2020-09-25

**Authors:** Bartali Ruben, Gaixia Zhang, Tong Xin, Speranza Giorgio, Micheli Victor, Gottardi Gloria, Fedrizzi Michele, Pierini Filippo, Sun Shuhui, Laidani Nadhira, Tavares Ana C

**Affiliations:** Fondazione Bruno Kessler, Center for Materials and Microsystems IRST Via Sommarive 18 38123 Trento Italy bartali@fbk.eu; Department of Physics, University of Trento Via Sommarive 14 Povo 38123 Trento Italy; Institut National de la Recherche Scientifique – Centre Énergie, Matériaux et Télécommunications 1650 Blvd. Lionel–Boulet Varennes QC J3X 1S2 Canada; Department of Biosystems and Soft Matter, Institute of Fundamental Technological Research, Polish Academy of Sciences ul. A. Pawinskiego 5b 02-106 Warsaw Poland

## Abstract

Graphene-based materials have demonstrated high chemical stability and are very promising for protection against the corrosion of metal surfaces. For this reason, in this work, protective layers composed of graphene oxide, reduced graphene oxide and their mixtures were investigated, respectively, against the corrosion of the surface of lead induced by water drops. The materials were deposited on a Pb surface from their suspensions using a Meyer rod. The surface chemical composition, morphology and structure of the coatings were studied by X-ray photoemission spectroscopy (XPS), scanning electron microscopy (SEM), atomic force microscopy (AFM) and stylus profilometry. Moreover, a specific methodology based on the evolution of the water contact angle with time was used to evaluate the reactivity of the lead surface. The results show that the graphene-based materials can form an efficient barrier layer against the degradation of the Pb surface and that the degradation process induced by water is reduced by more than 70%. Moreover, unexpectedly, the best protective performance was obtained using graphene oxide as the coating.

## Abstract 2

Graphene-based materials have demonstrated chemical stability and are very promising for protection against the corrosion induced by water on lead surfaces.

## Graphical abstract

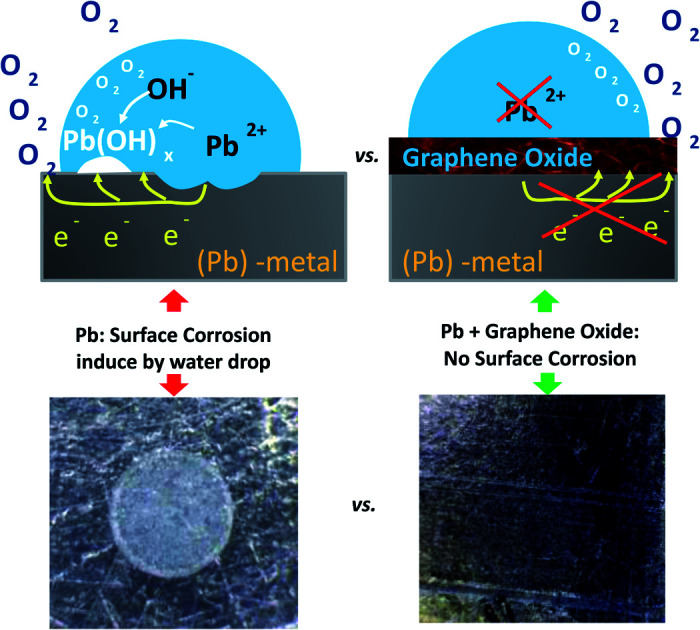

## Introduction

Lead was used in antiquities, jewelry, water pipes, or for the lamps in buildings. During the Roman era, Pb was used in plumbing systems. During the mediaeval period, lead was used as a roofing material, and the Padua Dome with its characteristic white color is a very nice example.^1,2^ Nowadays, Pb, which is still an essential material, is widely used in lead–acid batteries, which are still a practical solution for energy storage in smart grid systems due to their low cost and high efficiency.^3,4^ Lead is also used (i) as the electrode material for the electro-reduction of carbon dioxide in aqueous solutions, (ii) as a component to realize piezoceramic devices, (iii) to realize materials with a perovskite structure and (iv) or in soldering technologies.^5–14^ Moreover, thanks to its high density, it is widely used to realize shielding against sound or X-ray radiation.^10^ Metallic lead is abundant and malleable. It also shows excellent corrosion resistance when exposed to air, soil, freshwater, and seawater.^2^ However, in contact with water from moisture or with water drops which contain a high gradient of dissolved oxygen, the corrosion process is accelerated.^15^ This electrochemical corrosion phenomenon is described by the following equation:

1 2Pb(solid)+ O_2_(gas) + 2H_2_O(liquid) → 2 Pb(OH)_2_(solid)

In addition, the change of pH due to the gradient of oxygen in water accelerates the metal corrosion, as reported by DeGrutyer.^16^ The lead ions dissolved in water react with hydroxyl groups to form lead hydroxide or react with dissolved carbon dioxide to form lead carbonate.^17^ Due to the hazardous nature of these lead compounds and the negative effect of corrosion on the visual aspect of artifacts, protection of the lead surface is needed.^1,18,19^ Moreover, the protection of lead surfaces against the formation of dangerous compounds is expected to help in the handling of lead products in daily life and to preserve the aesthetics of historical artifacts like coins and statues.^20^ Therefore, it is essential to investigate how to inhibit the formation of lead compounds when the surface is wetted by water drops. Various deposition methods can be used to protect the metal surfaces such as plasma deposition, evaporation, and atomic layer deposition.^21–25^ Atomic layer deposition, in particular, can cover the surface of the metal with consecutive atomic layers in a very conformal mode. This results in excellent protection of the surface against corrosion.^26^ Unfortunately, all these techniques require vacuum technology or dangerous chemical precursors, and they are costly and need a specific implant. A promising alternative technology to protect metal surfaces is the use of inks and paints based on graphene and graphene oxide (GO).^27–29^ The layers of these 2D materials can follow the form of surfaces similarly to phyllo dough and provide protection against chemical agents. Su *et al.* demonstrated that a reduced graphene oxide (rGO) coating can act as a practically perfect barrier blocking all liquids, gases, and chemicals such as Cl^−^ ions.^27^ Singh *et al.* proved that a rGO – polymeric isocyanate cross-linked composite coating on copper has excellent resistance to Cl^−^ (chlorine) ions.^30^ Chen *et al.* demonstrated the passivation effect of graphene coating on Cu and a Cu/Ni alloy.^31^ Similar results were found by Topsakal *et al.* on Al(111).^32^ Schriver *et al.* also observed similar results but only in a short term (weeks); in fact, they observed that the graphene layer can promote the corrosion of the Cu surface in a long-term test (months).^33^ Lu *et al.* partially overcame this problem by passivating the graphene defects using ALD.^32^ Finally, Mayavan developed a graphene ink to inhibit corrosion of iron in a chloride environment.^34,35^ Most of the previous studies are focused on the barrier proprieties of graphene materials against corrosion of metal surfaces in an acidic environment. Nevertheless, in daily life, the main origin of surface degradation is water, *e.g.* copper corrosion in solar collectors.^36^ Water can degrade the surface of a wide number of materials, for isntance by the differential aeration corrosion process, water adsorption and ionic diffusion and exchange process, and hydrolysis induced by absorbed water, can degrade the surface of a wide number of materials.^15,37–45^ Recently Wang B. *et al.* reported interesting work on the barrier properties of graphene against water-induced degradation. The authors successfully used the impermeable barrier properties of graphene against water-induced degradation of silicate glasses.^46^ However, no work reported in the literature reports on: (i) the corrosion of lead induced by water drops and (ii) the effect of graphene and graphene-based materials as a protective barrier of lead surfaces. As a result, we developed protective layers deposited on Pb using a simple method based on graphene inks and the use of a Meyer rod. Moreover, we developed an affordable method based on contact angle measurements to determine the reactivity of water drops with lead and the protective properties of the deposited layers. Graphene-based inks are formed by GO, rGO, mixtures of GO and rGO flakes in 2-propanol (2-propanol is a low-cost and safe alternative to toxic solvents).^47^ Suspensions were obtained by mixing different percentages of GO and rGO. In the first part of this work, we estimate the effect of water drops on the surface chemistry and reactivity of lead using a dynamic contact angle profilometer and X-ray photoelectron spectroscopy. In the second part, we discuss the protective effect of the GO/rGO layers on lead surfaces against water corrosion.

## Results and discussion

### Evaluation of the reactivity of lead surfaces using the dynamic contact angle

**Fig. 1:**
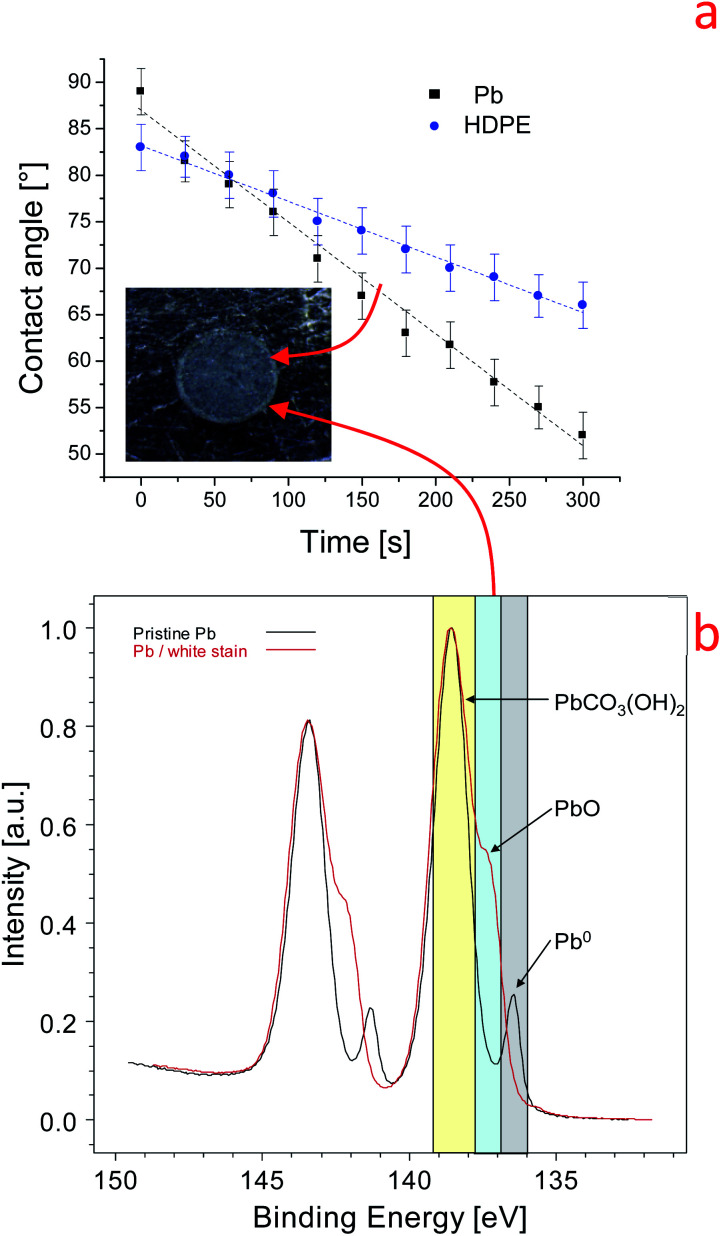
(a) Variation of the dynamic contact angle on lead and HDPE. Inset: optical microscope image of the lead surface at the end of the experiment. (b) High-resolution Pb 4f spectrum of the pristine lead surface (black line) and of the white stain on the lead surface (red line).

To investigate the reactivity of the Pb surface with water drops, two strategies were utilized in this work: (i) variation of the water contact angle as a function of time (dynamic contact angle) and (ii) estimation of the volume of lead salts using water drop/lead interaction. Dynamic contact angle measurement is a powerful method to study the evolution of liquid–surface interactions.^48^ The variation of the water-contact angle over time can be due to the reaction of water with the metal and to the evaporation of water. Moreover, the evaporation of water can affect the contact angle value because a drop is only 2 microliters. To understand the effect of evaporation on the dynamic contact angle values, we first studied the evolution of the contact angle on inert materials with different wettabilities: polydimethylsiloxane (PDMS, 104°), high-density polyethylene (HDPE, 83°), SiO_2_ (44°) and Si (68°). The tests were conducted at 21.5 °C and a relative humidity of 27.5%. The trend found for the contact angle (WCA) on HDPE is reported in Fig. 1a (blue dots). A monotonic reduction of the contact angle with time was observed, and this trend can be fitted using a linear equation in the range between 0 and 300 seconds (reactive range):

2 Contact angle (time) = *a* + *b* (time)

where *a* is the intercept of the fit and *b* is the slope of the straight line. The intercept *a* gives the intrinsic contact angle of water with the surface without any effect of evaporation. The slope *b* measures the rate of the variation of the contact angle with time, which can be due to evaporation of the water drop and to variations of the surface chemical properties because of water surface-reactions (contact angle surface reactivity). On inert materials where only evaporation of water occurs, we observed that the value of slope *b* is always lower than −0.06° min^−1^ (*e.g.*, −0.052° min^−1^ on silicon). PDMS shows the lowest *b* value (−0.03° min^−1^), while HDPE shows the highest *b* value (−0.056° min^−1^). The dynamic test was then conducted on the Pb surface, as shown in Fig. 1a (black squares). The starting contact angle on Pb is 87° which is similar to the one found for HDPE, but the time evolution of this contact angle is much faster (*b* = −0.12 ± 0.04° min^−1^) than that for HDPE and the other inert surfaces (2.2 times higher). As depicted in Fig. 1a, after the drop is evaporated, a white stain remained on the Pb surface due to differential aeration corrosion and precipitation of the corrosion products. This was confirmed by XPS analysis of the white stain, as shown in Fig. 1b. The Pb 4f peaks for the metal (136.8 eV), carbonate (138.3 eV) and Pb(CO)_3_(OH)_2_ (138.7 eV) forms are recognized. Consistently, the C 1s core line shows two main components, at 285 eV related to the C–C bond and at 288.5–289.9 eV related to O–C=O bonds (Fig. S1†).^49–52^

We can reasonably conclude that the high water contact angle on the pristine Pb surface is induced by carbon airborne contamination (285 eV), in agreement with what has been reported on graphite surfaces^48,53^ (S6). Further, the XPS findings in Fig. 1b indicate that water induces the transformation of Pb metal into Pb hydroxide and oxide according to eqn (1).^15^ The water–lead interaction promotes the formation of oxides, hydroxides, and hydrocarbonates which are more polar than the airborne contaminations. This process increases the surface tension and the hydrophilicity of the Pb surface. The variation of the physical–chemical properties of the Pb surface is manifested by the time dependence of the dynamic contact angle, as shown in Fig. 1a. For this reason, we named the *b* slope of eqn (2) the contact angle surface reactivity (hereafter named C.A surface reactivity) based on water contact angle measurements.

### Reduced graphene oxide/graphene oxide coatings on lead

**Fig. 2:**
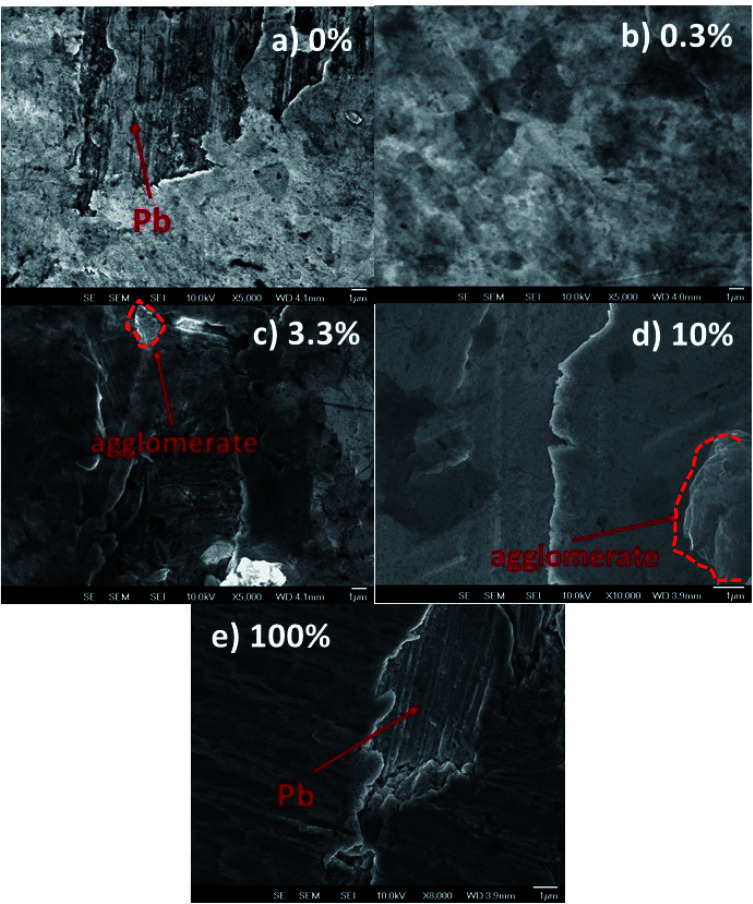
SEM images of Pb surfaces coated with reduced graphene oxide (rGO)/graphene oxide (GO) with different compositions. The content of rGO in the ink is: (a) 0%, (b) 0.3%, (c) 3%, (d) 10%, and (e) 100%. The balance wt% is GO. In (a) and (e) the morphology of the pinholes is highlighted to show the difference between the morphologies of the coatings and the pristine Pb surface.

The Pb surfaces were coated with graphene-based flakes using a Meyer rod. The average thickness of the coatings is 20–30 nm. From a macroscopic point of view, all coatings covered the substrate almost completely. However, scanning electron microscopy images revealed pinholes or the agglomeration of graphene nano-flakes at the micro and nanoscale levels. Fig. 2 shows the scanning electron microscopy images of different coatings obtained with 0%, 0.3%, 3.3%, 10%, and 100% rGO inks. Fig. 2a and e shows the pinhole regions on the coating prepared with 0% rGO and 100% rGO, to highlight the difference in morphology between the lead surface and the graphene-based coatings. Even if some pinholes are present on the surface (quite common for this kind of deposition method and without the use of a cleanroom), the layers prepared using rGO and GO are, in general, compact and without agglomerates. Fig. 2c and d shows the morphologies of the coatings containing 3.3% and 10% rGO, and creeps and agglomerated flakes are present on the surface. A similar result has been obtained on a flat silicon substrate using AFM, Fig. S3 and S4.† This indicates that the observed differences in the coating morphology are not induced by the morphology of the substrate but are related to the intrinsic coating structure induced by the different chemical compositions of the graphene based inks. The formation of agglomerates in the samples indicates that rGO does not disperse well in isopropanol, and the effect of compression using a Meyer rod is too low to obtain a fully homogeneous coating. The inadequate dispersion of graphene in isopropanol and isopropanol/GO solution is due to the high polarity of the solvent that probably induces a preferential dispersion of GO in isopropanol. The large agglomerates of graphene flakes are prone to detachment, exposing the surface of lead to external agents such as water drops.

**Fig. 3:**
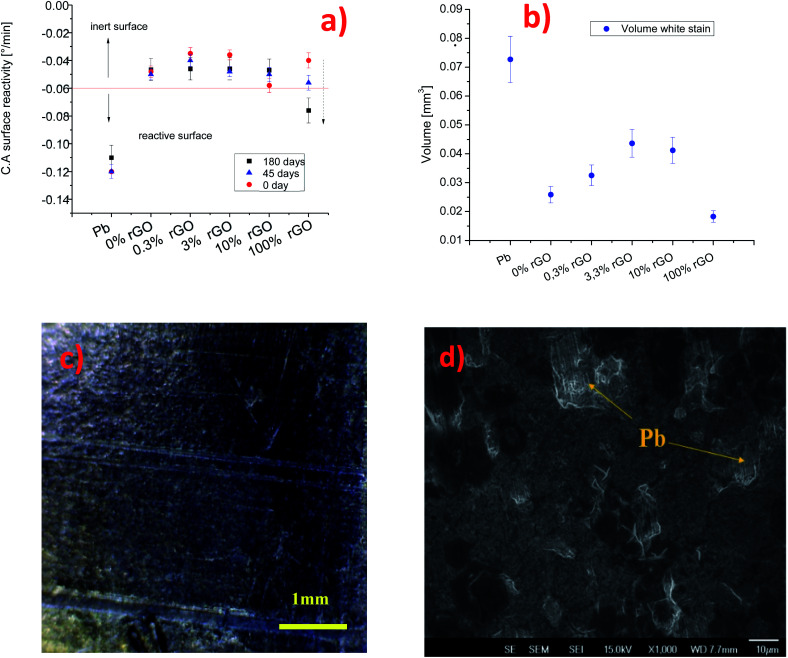
Variation of (a) the C.A surface reactivity and (b) white stain volume on the lead surface coated with rGO layers, (c) optical image of 100% GO coatings and (d) SEM image of lead coated with 100% rGO after 6 months of ageing.

To evaluate the barrier properties of the coatings, we cross-checked the results obtained by dynamic contact angle measurements, profilometry and SEM. As shown in Fig. 3a, the C.A surface reactivity of the lead surface with water is strongly suppressed when the surface is covered by the graphene-based inks. The pure GO coating shows an intrinsic contact angle of 44° and *b* = −0.048° min^−1^. For the sample prepared from 0.3% rGO ink, the intrinsic contact angle is less than 20° and the C.A surface reactivity parameter is −0.03° min^−1^, while for 10% rGO the contact angle is 60° and the C.A surface reactivity is −0.058° min^−1^. For the samples using 100% rGO, the water contact angle is 80° while *b* is lower than −0.04° min^−1^. The less effective barrier is the one formed from the 10% rGO ink. To obtain a semiquantitative appraisal of the extent of corrosion of the lead surface, we estimate the volume of the stain on the samples using a stylus profilometer. The average thickness and width of the white stain are used to approximately estimate the stain volume, S5. The volume of the white stain on lead (inset in Fig. 1a) is 0.07 mm^3^, whereas on the sample covered with 0% GO (100% rGO) it is only 0.025 mm^3^, 2.8 times less compared to that of bare Pb. The volume of the white stains on other coated surfaces varies between 0.03 and 0.045 mm^3^, and it tends to increase with the rGO content in the ink. Meanwhile, the stain's volume on the coating based on 100% rGO is only 0.018 mm^3^, which is the lowest value measured (3.8 times lower than that on lead) and is almost imperceptible, Fig. 3c. The capability of the coatings to protect the lead surface against water drop corrosion was tested again after 45 and 180 days (“long-term test”). Fig. 3a compares the C.A surface reactivity parameters with those determined on fresh samples (0 days). After 45 days the *b* value of the coating based on 100% GO is similar to the one determined for the fresh sample (−0.05° min^−1^). Instead, for coatings containing rGO, a sensible increase of the C.A surface reactivity is observed. Still, in all cases, the *b* value is lower than −0.06° min^−1^, and therefore within the range of “inert” surfaces. The C.A surface reactivity parameter of samples aged for 180 days further increases, as shown in Fig. 3a, but only slightly for coatings containing GO and significantly for the 100% rGO coating (−0.076° min^−1^). This value is between the values for pristine Pb and the values for inert surfaces. This indicates that the reactivity of the 100% rGO surface increased with aging, which means that this coating tends to lose its barrier properties. SEM analysis confirmed that after 6 months the rGO film detached from the Pb surface. An evident delamination process is shown in Fig. 3d.

We emphasize that the results reported here are related only to the protective properties of the coatings against water drops (differential aeration corrosion). The results do not reflect the protective properties of the coating when the sample is fully immersed in water, where the mechanism of corrosion could be different.

**Fig. 4:**
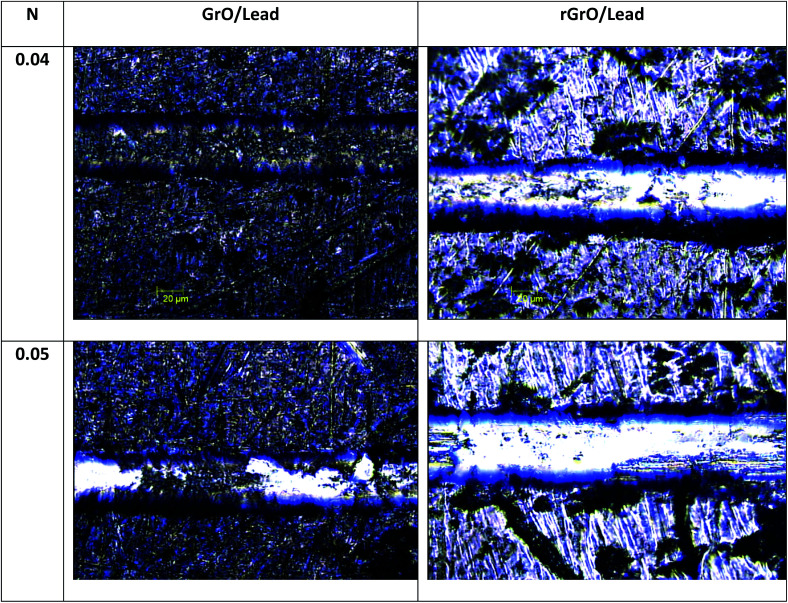
Scratch resistance of GO coatings and rGO on lead using the micro scratch test with loads of 0.04 N and 0.05 N.

These findings are in agreement with the results reported in the literature on the long term protective properties of graphene.^33,54^ Both studies underlined that graphene can induce degradation of the metal surface by galvanic corrosion. This could be a probable mechanism, but we emphasize that in our case the reduction of the protective properties of the 100% rGO coating is accelerated by poor adhesion of rGO to the lead surface. As in graphite, graphene-like layers can interact exclusively through van der Waals forces which are weaker than the polar interactions typical of GO.^55^ As shown in Fig. 4, the micro-scratch test results have confirmed these observations. The GO coating tends to delaminate using a load of 0.05 N, while rGO tends to delaminate at 0.03 N. Instead, the coatings composed of mixtures of GO and rGO are stable with good protective properties even after 6 months of aging. As mentioned above, the coating with 100% GO is the most stable one and this could be due to better adhesion to the lead surface compared to other films. The better adhesion of GO promotes good mechanical stability of the coating on the lead surface. Moreover, GO is an insulating material and therefore is able to inhibit the galvanic corrosion of the Pb surface as suggested by Cui *et al.*^54^ We remark that the protective properties of the “fresh” nanocomposite films (fabricated from the mixtures of rGO and GO) are not the average of the protective proprieties of the coatings fabricated from the two single materials. In general, the nanocomposites perform worse than pure reduced graphene and pure graphene oxide coatings. Nevertheless, the presence of graphene oxide in the nanocomposite improves the barrier properties and stability of the films with respect to the coatings formed using only reduced graphene, supporting the observation and the indications of Cui *et al.*^54^ We remark that this study is limited to graphene-based coatings, and we believe that a comparison with other kinds of coatings and deposition techniques is necessary to have a more clear techno-economical assessment of these thin films. Moreover further corrosion analysis is necessary to understand in a quantitative manner the protective properties of GO and rGO coatings.

## Experimental

### Synthesis of the graphene materials

The GO was synthesized by a modified Hummers method. Graphite powders were oxidized using potassium permanganate in a mixture of concentrated sulfuric acid and nitric acid at 80 °C. After ultrasonication and concentration processes, the GO was washed with DI water several times. Finally, the GO powders were dried at 80 °C for 24 h. Subsequently, reduced graphene was obtained from GO by an explosion–expansion method and thermal reduction at 1050 °C.^56^ All chemicals were purchased from Sigma-Aldrich. The reduced graphene oxide C 1s spectrum is mainly composed of carbon–carbon bonds (284.4 eV, >77% relative at.con),^48,53,57,58^ Fig. S1.† The C 1s spectrum of GO is composed of carbon–carbon bonds (284.4 eV, 30 at%), C–OH groups (286.8 eV, 30.1 at%) and C=O groups (287.8 eV, 7 at%), Fig. S1.† The Raman spectra show the characteristic 1D, 1G, and 2D peaks and the ratio *I*_D_/*I*_G_ is 1.042 for GO and 0.93 for rGO, Fig. S2.†

### Preparation of the graphene coatings

The lead substrates (Nuclead, chemical grade, 1/16 inch thickness) were cleaned using sandpaper 1200, immersed in isopropyl alcohol (99.9% Sigma Aldrich) and treated in an ultrasonic bath for 10 min. The samples were dried using nitrogen gas (99.999999%) for 1 minute. The graphene inks were obtained by dispersing 3 g of rGO and GO flakes in 5 ml of isopropanol. The wt% of rGO in the inks was varied as follows: 100%, 10%, 3%, 0.3% and 0%. GO has a complementary wt%. The samples are named according to the percentage of rGO: 100% rGO, 10% rGO, 3% rGO, 0.3% rGO, and 0% rGO. A Meyer rod was used to spread the inks on the surface and to improve the homogeneity of the graphene layers. To reduce the presence of grooves in the coating, a spacer with a thickness of 25 microns was placed between the lead surface and the Meyer rod.

### Physicochemical characterization

The surface chemical properties of the graphene materials was analyzed by X-ray photoelectron spectroscopy (Axis DLD Ultra – Kratos UK) with a monochromatic mono-Al source. The Pb^0^ peak at 136.8 eV was used as the reference line to calibrate the positions of other spectral lines. Spectral features were analyzed using RXPS homemade software based on the R© platform. Shirley background subtraction and Gaussian components were used for spectral fitting. The morphology of the coatings was analyzed by scanning electron microscopy (SEM, JEOL JSM-7401F). The coatings thickness and roughness were measured using a profilometer (KLA Tencor P6). Raman spectra and atomic force microscopy (AFM) topographies were collected using a hybrid AFM/OT/Raman microscope described in more detail elsewhere.^59^ The instrument was equipped with an Olympus MPlan N objective (numerical aperture of 0.75× and 50× magnification) used for focusing a laser on the sample and a computer-controlled high-precision piezo stage. Measurements were performed on a silicon wafer placed on an Olympus IX71 inverted optical microscope coupled with the hybrid equipment. Raman measurements were carried out using a double-frequency Nd:YAG laser with a wavelength of 532 nm and an energy of approximately 2.33 eV (NL202, Ekspla) as the excitation source. The AFM microscope was equipped with a conical tip shaped cantilever (HA_NC, NT-MDT) with a typical force constant of 12 N m^−1^ and a curvature radius of 10 nm, which was operated at its resonance frequency around 235 kHz. The images were evaluated using Nova software.^59^ The wetting properties of the surfaces were studied using the temporal evolution of the sessile contact angle with deionized water (18 MΩ), and hereafter named the dynamic contact angle. In this work, 2 μL drops of deionized water were placed on the sample's surface. The images were acquired with a CMOS camera and drop-analysis was conducted afterwards.^60,61^ The measurements were conducted in the laboratory with a controlled temperature of 23 °C and with 50% average relative humidity. The microstratch tests were performed using a Micro-scratch test instrument (CSM, Switzerland) equipped with a diamond tip of 200 microns in diameter.

## Conclusions

In this work, the protection of lead against the corrosion induced by water drops using graphene-based materials as protective barriers has been investigated. Films based on GO and rGO were deposited on Pb surfaces from inks with different GO and rGO contents and using a Meyer rod. The sessile water contact angle, XPS, SEM and profilometry were used to investigate the barrier properties of the coatings. A new methodology based on the dynamic water contact angle was developed and successfully used to study the C.A surface reactivity of lead surfaces. The results show that all films were able to reduce corrosion by water drops significantly. The best results on fresh samples were obtained with films realized from 100% rGO and 100% GO, and this was explained in terms of more uniform and defect-free surfaces. The impact of the aging of the coatings on their barrier properties was evaluated for up to 6 months. In the long term (6 months), the 100% rGO film showed a sensible reduction of its protective effect. This was due to the delamination of the reduced graphene layer from the Pb surface. Instead, the films based on GO were more stable upon aging, indicating that layers based on graphene oxide are preferable to protect Pb surfaces against differential aeration corrosion induced by water drops.

## Conflicts of interest

There are no conflicts to declare.

## Supplementary Material

NA-002-D0NA00212G-s001
