# Supplementary material for: Graphene oxide/reduced graphene oxide films as protective barriers on lead against differential aeration corrosion induced by water drops
Source: Nanoscale Adv. 2020 Sep 25;2(11):5412–20. doi: 10.1039/d0na00212g (PMC9419162; doi:10.1039/d0na00212g)
Supplement: NA-002-D0NA00212G-s001 [file NA-002-D0NA00212G-s001.pdf]

Electronic Supplementary Material (ESI) for Nanoscale Advances.
This journal is © The Royal Society of Chemistry 2020

Supporting Information

Graphene oxide / reduced graphene oxide films as protective barriers on lead against surface corrosion induced by water drops

R. Bartali [1,2], G. Zhang [3], X. Tong [3], G. Speranza [1], V. Micheli [1], G. Gottardi [1], M. Fedrizzi,[1], F. Pierini[4], S. Sun [3], N. Laidani[1] and A.C. Tavares[3]

[1]Fondazione Bruno Kessler, Center for Materials and Microsystems IRST, Via Sommarive 18, 38123 Trento, Italy.

[2]Department of Physics, University of Trento, Via Sommarive 14 Povo, 38123 Trento Italy.

[3]Institut National de la Recherche Scientifique – Centre Énergie, Matériaux et Télécommunications, 1650 Blvd. Lionel–Boulet, Varennes, QC, J3X 1S2, Canada.

[4]Department of Biosystems and Soft Matter, Institute of Fundamental Technological Research, Polish Academy of Sciences, ul. A. Pawinskiego 5b, 02-106 Warsaw, Poland.

S1 XPS, Raman and AFM analysis of graphene oxide (GO) and reduced graphene oxide (rGO) deposited on a flat silicon wafer

To study the intrinsic surface chemical properties of graphene-based materials the GO and rGO were deposited on a flat silicon wafer (thickness 20-30nm). The reduced graphene oxide C 1s spectrum is mainly composed by carbon-carbon bonds (284.4 eV, >77% relative at.con) [39],[40],[41]. The C 1s spectrum of GO is composed by carbon-carbon bonds (284.4 eV, 30 at.%), C-OH groups (286.8 eV, 30.1 at%) and C=O groups (287.8 eV, 7 at%), **Figure S1**.

The Raman spectra are reported in **Figure S2**. In both cases prominent D and 1G peaks and the 2D band are observed: D band (~1350 cm-1), G band (~1580 cm-1) and the 2D band (~2700 cm-1). The I_D/I_G ratio is 1.042 for GO and slightly lower for rGO (0.93).The morphology of the GO and rGO flakes was investigated by AFM, **Figure S3**. The coating layer with 100% of GO appears quite compact, and the border of the flakes are well recognizable, **Figure S3a**. The dimension of the flakes is in the range of few microns. The edge appeared separated. This phenomenon tends to increase the roughness of the GO deposit that is 2.45 nm. The coating based on 100% rGO appears very smooth , **Figure S3e**. The average value of roughness is less than 1 nm. The samples based on the mixture of GO/rGO show a much more complex morphology. The films are formed by flakes of GO covered by the island of rGO, **Figure S3b, c and d**. In S3 b (0,3% rGO) the diameter of the rGO islands is around 50 nm, whereas in S3 **d (10% of rGO)** the surface of GO flakes is covered by the island of rGO with a diameter of 200 -250 nm. The graphene islands are well distributed on the surface of the samples.

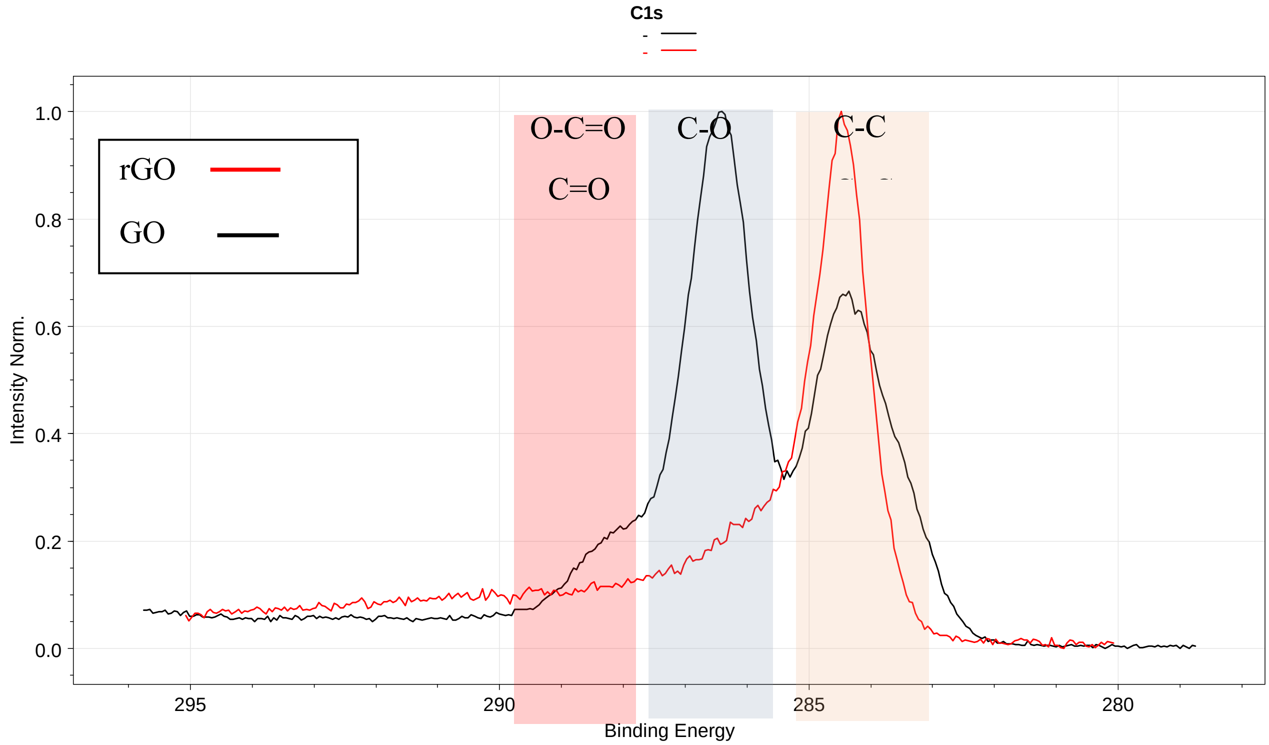

Figure S1. High-resolution C 1s spectra of graphene oxide (GO, black line) and reduced graphene oxide (rGO, red line) on a silicon substrate.

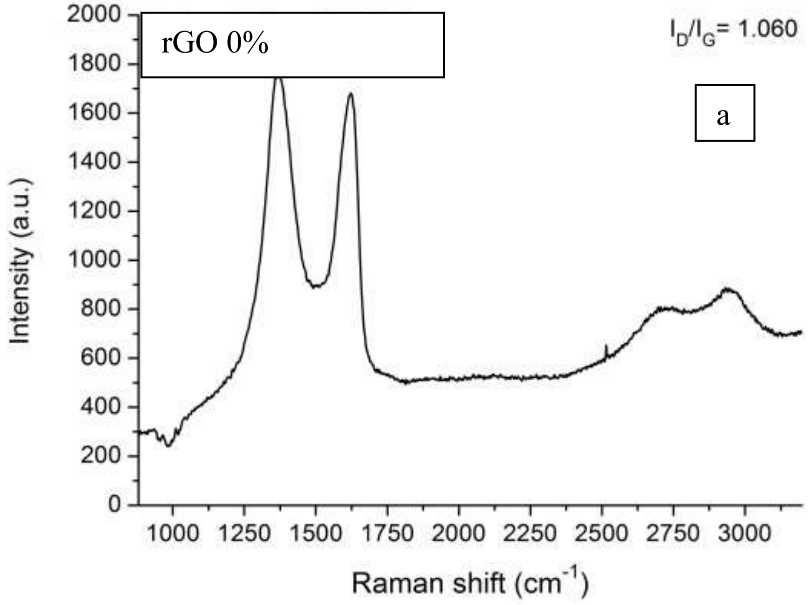

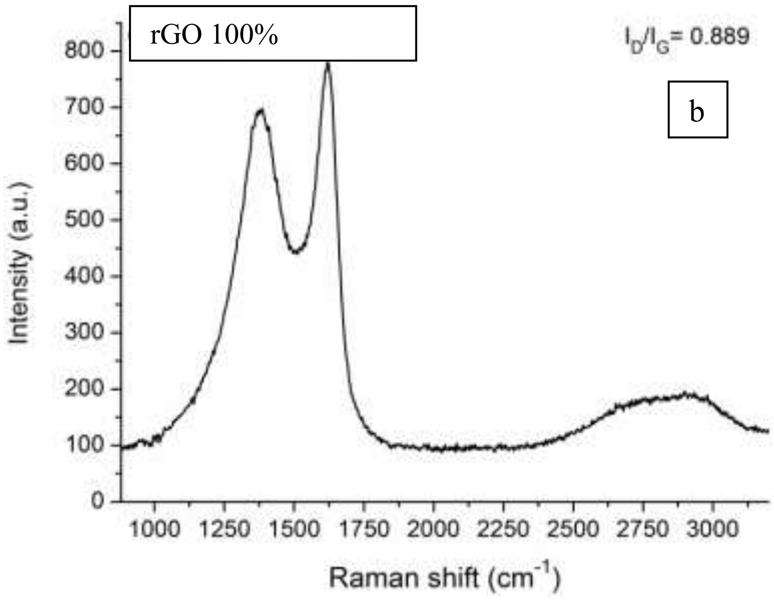

Figure S2. Raman spectra of silicon surface coated with **(a)** 0% rGO (100% GO) and **(b)** 100% rGO

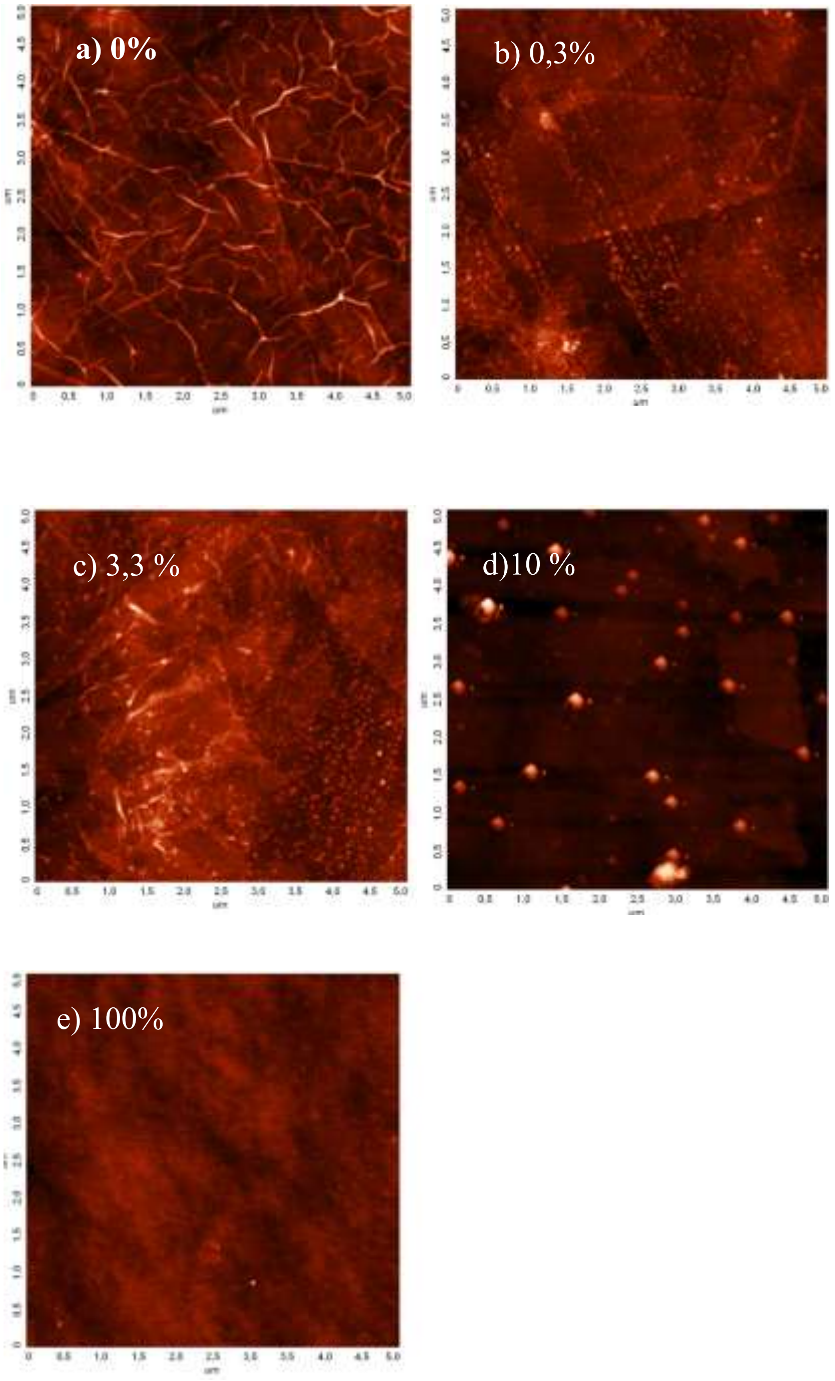

Figure S3. AFM images (topography mode) acquired on rGO / GO deposites on flat silicon substrates: **(a)** 0% rGO (100% GO); **(b)** 0.3 wt% rGO; **(c)** 3.3% rGO; **(d)** 10 % rGO; **(e)** 100% rGO.

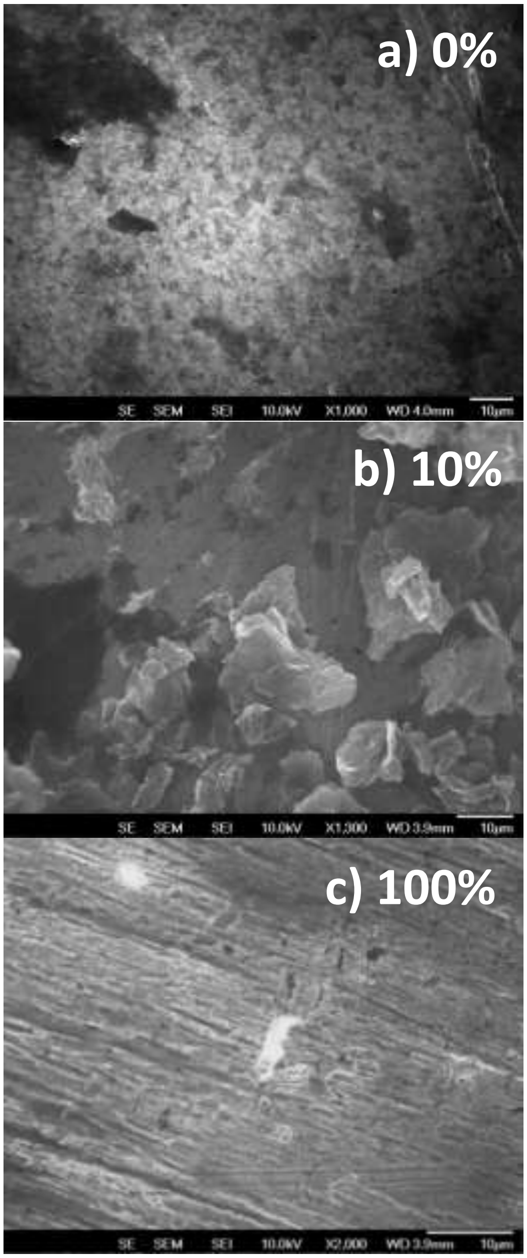

Figure S4. SEM images of the GO/rGO coating with different proportions on Pb: **(a)** 0.3 % rGO; **(b)** 10 % rGO and **(c)** 100% rGO.

S5 Estimation of volume of the white lead formation using stylus profilometry

The thickness and width of white stain formed onto Pb samples were measured using stylus profilometer. The measurements were repeated three times, and along with three different directions as shown in Figure S5, red line. Using a cylindric approximation of the shape of the white stain, the volume was calculated, e.g. for Pb 0.07 mm^3.

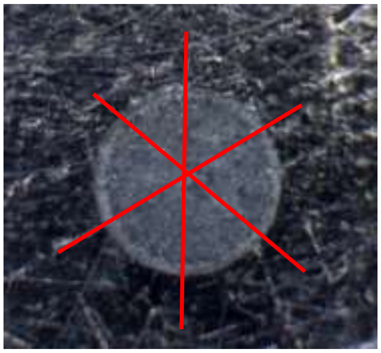

Figure S5. Picture of a white stain, the red line indicates the region where the profile on white stain was measured by a stylus profilometer.

S6 XPS on Lead white stain

The white stain and the unmodified lead surfaces were analyzed by XPS. The lead surface contains 32 at.% of carbon, mostly assigned to graphitic carbon from airborne contamination (C1s binding energy equal to 285.1 eV). The other minor components of the C1s peak are attributed to carbon species bonded to oxygen (C=O at 287.1 eV and O-C=O at 289.3 eV). On the white stain, the C1s peak shows a significant increase of the peak area for the component at 290 eV, typical of HO-C=O functional group.

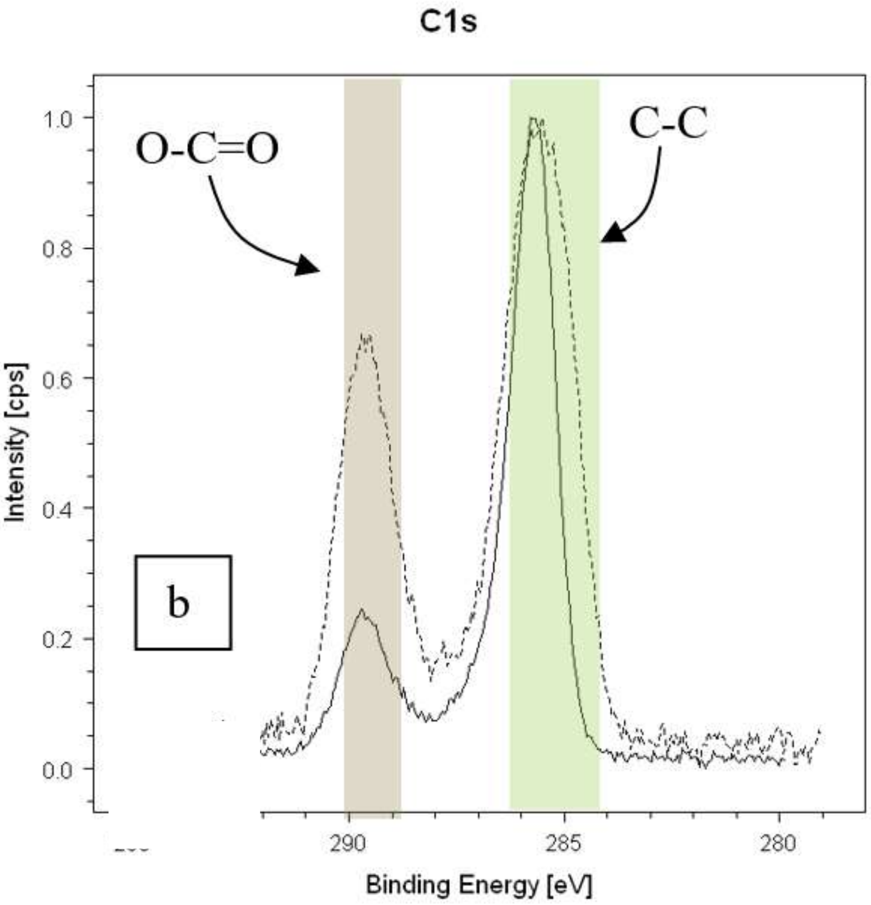

Figure S6 XPS (a) Pb 4f and (b) C 1s core line spectra for lead (straight line) and white stain (dotted line).
